# Effect of Presence and Concentration of Plasticizers, Vegetable Oils, and Surfactants on the Properties of Sodium-Alginate-Based Edible Coatings

**DOI:** 10.3390/ijms19030742

**Published:** 2018-03-06

**Authors:** Tugce Senturk Parreidt, Michael Schott, Markus Schmid, Kajetan Müller

**Affiliations:** 1 Technical University of Munich, TUM School of Life Sciences Weihenstephan, Chair of Food Packaging Technology, Weihenstephaner Steig 22, 85354 Freising, Germany; schmid@hs-albsig.de; 2 Fraunhofer Institute for Process Engineering and Packaging IVV, Giggenhauser Straße 35, 85354 Freising, Germany; michael.schott@ivv.fraunhofer.de (M.S.); kajetan.mueller@hs-kempten.de (K.M.); 3 Faculty of Life Sciences, Albstadt-Sigmaringen University, Anton-Günther-Str. 51, 72488 Sigmaringen, Germany; 4 Faculty of Mechanical Engineering, University of Applied Science Kempten, Bahnhofstraße 61, 87435 Kempten, Germany

**Keywords:** edible coating, sodium alginate, plasticizer, vegetable oil, surfactant, surface tension, coating stability

## Abstract

Achieving high quality of a coated food product is mostly dependent on the characteristics of the food material to be coated, the properties of the components in the coating solution, and the obtained coating material. In the present study, usability and effectiveness of various components as well as their concentrations were assessed to produce an effective coating material. For this purpose, different concentrations of gelling agent (sodium alginate 0–3.5%, *w*/*w*), plasticizers (glycerol and sorbitol (0–20%, *w*/*w*), surfactants (tween 40, tween 80, span 60, span 80, lecithin (0–5%, *w*/*w*), and vegetable oils (sunflower oil, olive oil, rapeseed oil (0–5%, *w*/*w*) were used to prepare edible coating solutions. Formulations were built gradually, and characteristics of coatings were evaluated by analyzing surface tension values and its polar and dispersive components, emulsion droplet size, and optical appearance in microscopic scale. The results obtained showed that 1.25% sodium alginate, 2% glycerol, 0.2% sunflower oil, 1% span 80, and 0.2% tween 40 or tween 80 can be used in formulation to obtain an effective coating for hydrophobic food surfaces. Three formulations were designed, and their stability (emulsion droplet size, optical characteristics, and creaming index) and wettability tests on strawberry showed that they could be successfully used in coating applications.

## 1. Introduction

The development of new packaging materials for the food industries, particularly due to increased health and environment-consciousness, is a rapidly growing area. Hence, there has been an increased amount of research on renewable, sustainable materials to use for packaging [1]. One of these approaches is edible films and coatings, which are biodegradable packaging formulated from edible components such as various animal or vegetal origin substances [1,2]. Edible coatings and films have great advantage over conventional plastic packaging, as they can be a complete food coating or incorporated between food components such as baked pastry in a pie and ingredients in a pizza [1,3,4]. Edible coatings and films provide a fortification layer to decrease the mass transfer (water migration, gas transfer, migration of aromatic compounds and solvents, etc.) between the product and the surrounding medium, provide mechanical stability, and become a barrier against light [1]. Free-standing edible films have enough integrity that they can be cut and placed on food surfaces [5,6]. Conventionally, film solution is deposited on an inert surface, uniformly spread, and various techniques like solvent removal, thermal gelation, and melting followed by solidification are applied to obtain the stand alone wrapping material. On the other hand, edible coatings are applied on the surface of the food product by dipping, spraying, spreading, and vacuum impregnation methods, and are created as a thin layer on the surface [5,6,7].

The main gel-forming substances are classified according to their structural materials: hydrocolloids (i.e., proteins and polysaccharides), lipids, or a combination of them (i.e., composites) [2,8]. Among these, alginate is an extensively used polysaccharide that is quite abundant in nature. Alginate (alginic acid sodium salt) is a structural component in marine brown algae (Phaeophyceae, mainly Laminaria) and soil bacteria [9,10]. Food grade sodium alginate (E401) is affirmed as “Generally Recognized as Safe” (GRAS) and used as emulsifiers, stabilizers, thickeners, and gelling agents [11]. In Europe, alginic acid and its salts are listed as European Commission (EC)-approved additives [9,12]. Although alginate-based coatings produce rigid gel instantaneously in the presence of calcium or a bivalent ion, due to their hydrophilic nature, they exhibit poor water resistance [10,13]. As a rule of thumb, lipids are coating biopolymers, used for reducing water transmission. On the other hand, lipid-based films which contain hydrophilic polymers have durability and structural integrity [14]. Lipid elements can be included in the formulation to form composite coatings and to enhance the moisture barrier properties [3,15,16]. Lipids such as waxes (paraffin wax, beeswax, carnauba wax, candelilla wax, etc.), vegetable oil, mineral oil, acetylated monoglycerides, and sucrose esters of fatty acids can be used as coating materials [16,17].

Unplasticized coatings are brittle and not applicable for coating applications. Plasticizers such as glycerol, sorbitol, monoglycerides, polyethylene glycol, glucose, etc., are commonly used to overcome edible coating brittleness and improve flexibility and elongation of polymeric substances [2,16,18].

Surfactants are surface active agents, whose major characteristic is to be at higher concentration at the surface (liquid–solid, liquid–liquid, or liquid–air interface) than in the bulk of the liquid [19]. Based on the chemical structure of hydrophilic groups and the charge type of the surface active part, surfactants can be classified as anionic (negatively charged), non-ionic (no charged group), cationic (positively charged), and amphoteric (can be positively or negatively charged, or both, depending on the circumstances) [19]. Adhesion of the coating material to the surface of the product can be promoted by adding surfactants to the formulation due to reducing surface tension [3]. Sorbitan esters (spans) and their ethoxylates (tweens) are non-ionic surfactants with food approval [20,21]. Addition of multi-ether groups to the structure (ethoxylation) increases the water solubility of the surfactant. Moreover, water solubility feature increases with larger amounts of ethylene oxide [19]. They have many functional benefits: they can be also used as emulsifier, dispersant, and wetting and foaming agents [19,22]. They are stable over a wide pH range and they are electrolyte-tolerant [22]. Tweens are hydrophilic and are soluble or dispersible in water; on the other hand, spans are partly soluble in water [22]. Tweens are compatible with other surfactants and the synergistic effect between the surfactants are well known [19]. Phosphatidylcholine (PC), which are phospholipids with choline head group, is an important component of soybean lecithin and gives the natural surfactant characteristic to it [23]. Although the biggest concern about lecithin is its allergenicity, the Food Allergy Research and Resource Program (FARRP) at the University of Nebraska–Lincoln pointed out that soy lecithin does not contain sufficient soy protein residues to cause allergic reactions [24].

Functional properties and effectiveness of edible coating emulsions are strongly correlated with the wetting and uniform spreading ability of the coating on the targeted food product [25,26,27]. These concepts depend on the balance between adhesion (W_a_) and cohesion forces (W_c_), surface tension of the coating liquids, and surface characteristics (i.e., surface free energy) of the product [26]. Song and Springer introduced a digital-image-processing-based method to estimate the surface and interfacial tension of systems using the profile of a pendant drop [28,29]. However, determining the surface energy of the solids is not as straightforward as liquids. It can be measured indirectly with the help of various liquids with known values of surface tension and components. Additionally, there have been different theories to calculate surface free energy of a solid from contact angle data. One of these theories is the Owens, Wendt (1969), Rabel (1971), and Kaelble (1970) method (OWRK), which is based on a two-component model: polar and dispersive forces [30,31,32]. Similarly, polar and dispersive components of the liquid can be measured indirectly with the help of a solid with a well-known surface free energy and its components [33,34]. With the help of the contact angle created by the coating emulsion on the solid product surface, and surface tension of the coating emulsion, the wettability (W_S_) characteristic of the food product can be calculated [35].

Composition, preparation method, and the droplet size have strong effect on stability of the emulsions [36]. Generally, droplet sizes larger than 1 µm are affected by gravitational forces [36]. Creaming index (CI) values can be used to predict the behavior of the edible coating solution during storage [37].

Even though the effects of various edible coating formulations based on alginate gel matrix on quality parameters and shelf life of food products have been studied in detail, there has been less literature about the creation of the coating formula; the very initial step in alginate-based coating design was not extensively investigated and well-documented. Additionally, previous works have focused on a limited number of components in the design of coating formulations.

Therefore, the main aim of the present work was to design and optimize sodium-alginate-based edible coating formulations. This study investigated the formulation preparation step broadly and built the formulation gradually with experiments. Concurrently, the influence of the presence and the concentration of components to the physical properties (surface tension, as well as the polar and dispersive components) of alginate-based edible coatings were elucidated. Additionally, the relationship between surface tension and droplet characteristics of coating solutions were examined. Once the formulations were optimized, the stabilities of the formulated emulsions as well as the wettability characteristics on the selected food product (strawberry) were presented.

## 2. Results

### 2.1. Surface Tension of the Coating Solutions

**Figure 1:**
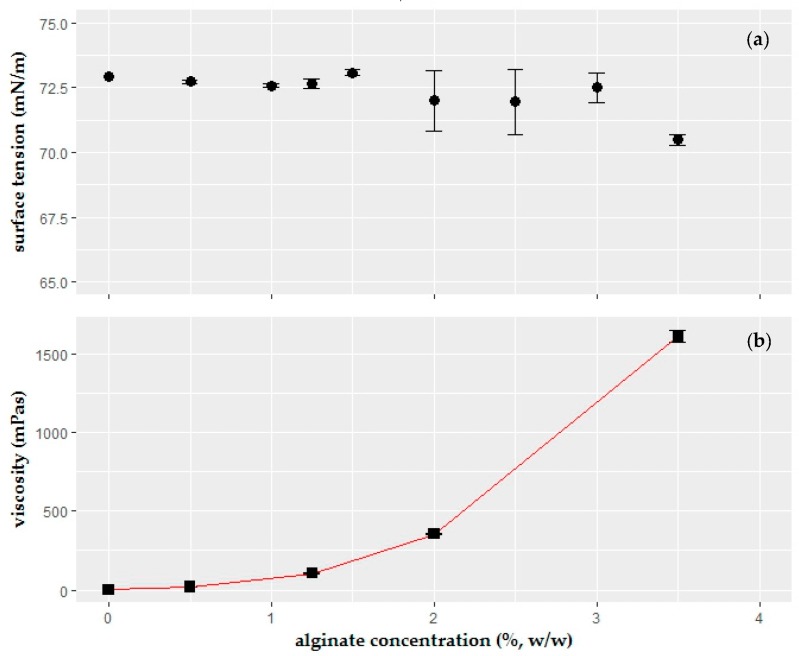
Effect of sodium alginate concentration (%, *w*/*w*) on (**a**) surface tension (mN/m); (**b**) viscosity (mPa∙s) of the coating solution (*n* = 3).

The effect of dissolved sodium alginate concentration on the surface tension of the solution is shown in Figure 1a. The results indicated a statistically insignificant decrease in surface tension when alginate concentration was increased (*p* > 0.05, Kruskal–Wallis test). However, the limiting factor, which determined the concentration of the alginate to be used in the formulation, was viscosity of the solution. Viscosity increased exponentially with the increased concentration, as shown in Figure 1b. Alginate concentration had a significant effect on the viscosity of the coating gel (*p* ≤ 0.05, Kruskal–Wallis test). The 3.5% (*w*/*w*) is selected as the highest concentration due to the high gel viscosity. According to the studies in the literature, coating thickness has been increasing proportionally with higher viscosity [38,39]. A thick coating on the food product is not a preferred feature in the coating process. The viscosity of the alginate solution increased drastically for the concentrations higher than 2%. On the other hand, a decent amount of gelling agent was required in order to achieve gel formation. Therefore, 1.25% (*w*/*w*) alginate was selected as the highest alginate concentration in the formulation with low effect of viscosity. Lower concentrations were not selected due to being able to observe the effects of alginate in the subsequent experiments.

**Figure 2:**
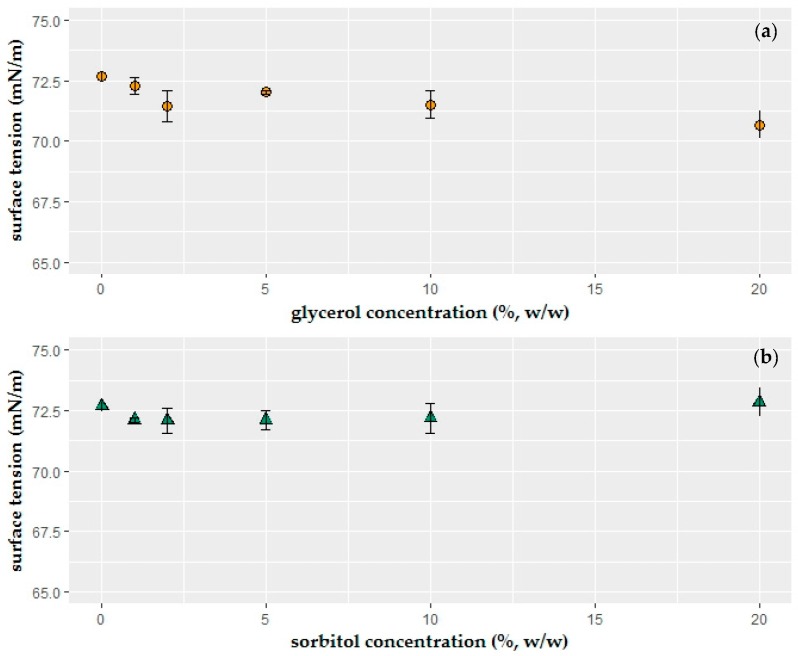
Effect of plasticizer (**a**) glycerol, (**b**) sorbitol concentration (%, *w*/*w*) on surface tension (mN/m) of the coating solution. Glycerol or sorbitol was incorporated into 1.25% (*w*/*w*) sodium alginate solutions (*n* = 3).

As a second step, the presence and influence of two different plasticizers (0–20%, 6 levels) on surface tension of the coating solution were investigated (Figure 2a,b). A two-way analysis of variance (ANOVA) test was run on the results. Both main and interaction effect analysis as well as TukeyHSD post hoc test showed that only 20% (*w*/*w*) glycerol-added solution had significantly different effects on surface tension values (*p* ≤ 0.05), and additionally there was no significant difference between glycerol and sorbitol. Sorbitol is a sweetener that is used to replace sucrose in the food products [40]. Hence, glycerol was chosen as plasticizer to obtain a natural taste in the edible coating.

Fresh-cut fruits and vegetables have high water activity on the surface. This characteristic enables the water-based edible coatings to spread easily on the coating surface, and water barrier properties should be taken into consideration in order to prevent water loss. Tapia, et al. [41] investigated the effects of glycerol concentration (1–2%, *w*/*v*) on barrier functionality of alginate-based edible coatings and concluded that glycerol concentrations higher than 1.5% (*w*/*v*) enhanced the water vapor resistance (WVR) of the alginate coating on papayas. Therefore, 2% glycerol concentration was selected for the formulation.

**Figure 3:**
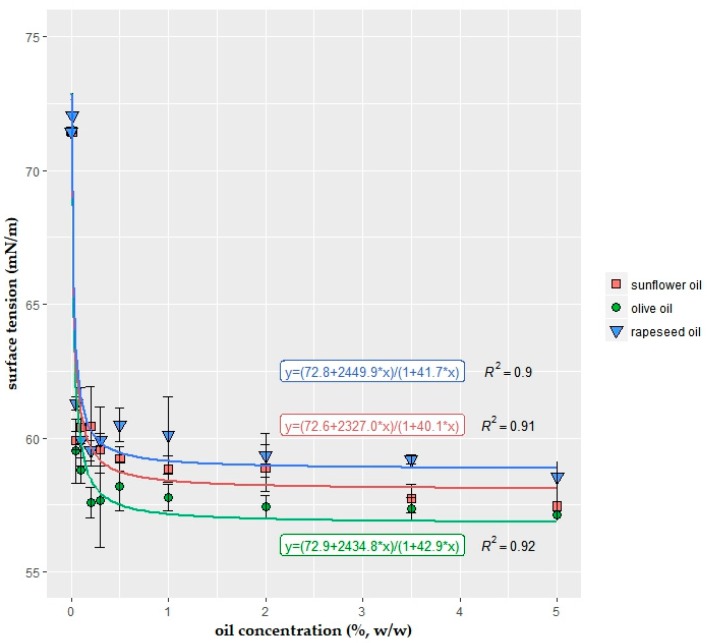
Variation of the surface tension (mN/m) of the solution with the concentration (%, *w*/*w*) of vegetable oils: sunflower, olive, and rapeseed oil. Each concentration was added into 1.25% sodium alginate + 2% glycerol solutions (*n* = 3 replications).

Vegetable oils were added as a lipid source to the 1.25% sodium alginate and 2% glycerol dissolved coating formulations. It was no surprise that oil did not reduce the surface tension drastically (Figure 3). Formulations of 100% sunflower oil, olive oil, and rapeseed oil had 32.68 ± 0.60 mN/m, 31.75 ± 0.25 mN/m, and 32.44 ± 0.27 mN/m surface tension, respectively. According to the separate Kruskal–Wallis tests results, oil type and concentration had a significant effect (*p* ≤ 0.05) on surface tension. Surface tension results of olive oil-added solutions were significantly different from sunflower- and rapeseed oil-added samples. The pairwise Wilcoxon rank sum post hoc test indicated that higher concentrations (>0.2% oil) do not significantly decrease the surface tension results.

The simplest fitting function to data points were found as rational function y = f(x) = (a + cx)/(1 + bx) for all three vegetable oils with fairly low number of variables. The goodness of fits was quantified by R^2^ (R-squared) values, which determine how close the data is to the fitted regression lines. The variables in the functions (a, b, and c) were very close, and goodness of fit values of the curves were high (R^2^ ≥ 0.90).

Despite the lower surface tension results, olive oil has certain drawbacks (i.e., having darker color compared to sunflower oil, being more expensive and having a strong, distinguishable odor and flavor). Due to the small differences in surface tension results, sunflower oil was selected as the lipid source for the formulation.

**Figure 4:**
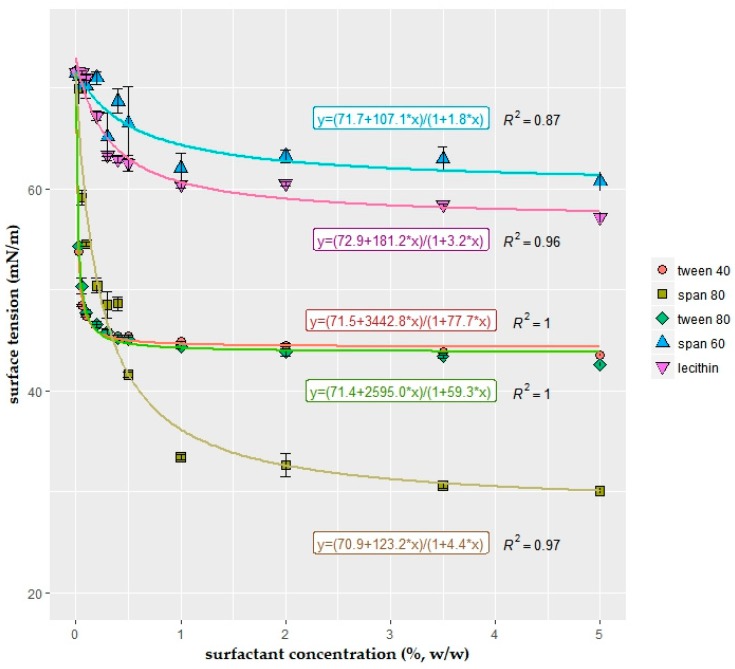
Variation of the surface tension (mN/m) of the solution with the concentration (%, *w*/*w*) of surfactants: tween 40 (polyoxyethylenesorbitan monopalmitate), tween 80 (polyoxyethylenesorbitan monooleate), span 80 (sorbitan monooleate), span 60 (sorbitan monostearate), and soy lecithin. Each surfactant concentration was incorporated into 1.25% sodium alginate + 2% glycerol solutions (*n* = 3).

Intact fruits and vegetables have low energy and hydrophobic surfaces. Porter [19] stated that non-ionic surfactants were adsorbed in higher amounts on non-polar or hydrophobic surfaces than polar surfactants. Therefore, non-ionic surfactants such as tween 40, tween 80, span 60, and span 80 were used in the present study. Additionally, soy lecithin was also included to the trials due to its emulsification effect and widespread usage in the industry. The effects of surfactant type and concentration on surface tension values are shown in Figure 4. Although the surfactants formed similar descending curves, the slopes were quite different. Yet, all reached their saturation point around 1%. Statistical evaluations showed that surfactant type, concentration, and their interaction had significant effect (two-way ANOVA, *p* < 0.05). For the lower surfactant concentrations (<0.5%), tween 40 and tween 80 were more effective in decreasing surface tension. However, for concentrations above 0.5%, span 80 was the most effective. The results showed that tween concentrations could be kept low; on the other hand, span 80 must be used in higher amounts (~1%) to reduce the surface tension to the utmost degree.

It was very interesting that the same rational function [y = f(x) = (a + cx)/(1 + bx)], which determined the descending curves of change in surface tension with increasing oil concentration (Figure 3), also constructed good fits to the surface tension versus surfactant concentration data points (Figure 4). The variable “a” is approximately the same for each equation (~71) due to starting at the same surface tension value in zero concentration (i.e., solutions containing only 1.25% alginate and 2% glycerol, without surfactant and oil addition). The “b” and “c” variables in the functions of tween 40 and tween 80 were very close to each other, as expected. However, it was found to be interesting that spans had also very similar “b” and “c” values despite being located quite far from each other in the graph. Apart from “c”, the change in the “b” value was particularly important in this distribution difference.

### 2.2. Polar and Dispersive Components of the Coating Solutions

**Figure 5:**
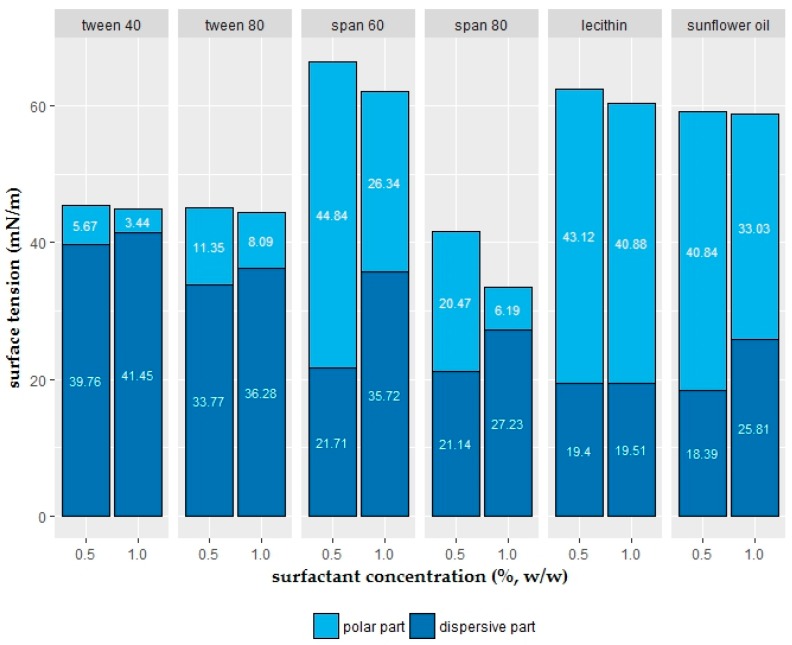
Effect of surfactant and oil type and concentration on polar (γ_L_^P^) and disperse (γ_L_^D^) part of the surface tension of coating solution. Each surfactant concentration was incorporated into 1.25% sodium alginate + 2% glycerol solutions (*n* = 3).

The calculated dispersive (γ_L_^D^) and polar (γ_L_^P^) components of surfactants (tween 40, tween 80, span 60, span 80, lecithin) and oil (sunflower) are presented in Figure 5. Surface energy and dispersive and polar components of the polytetrafluoroethylene (PTFE) film used in the calculation were 14.24 ± 0.52 mN/m, 14.18 ± 0.50 mN/m, and 0.06 ± 0.03 mN/m, respectively.

Error bars, showing standard deviations, were not generated due to the usage of mean values of all variables (i.e., contact angle, liquid surface tension, polar and dispersive components of PTFE film surface energy) in the calculations.

Polar forces of surfactants and sunflower oil decreased with increasing concentration. On the contrary, dispersive forces increased with the higher concentration. Besides this, dispersive forces of relatively more effective surfactants (i.e., tween 40, tween 80, and span 80) had higher values compared to their polar counterpart. On the other hand, relatively less effective surfactants and sunflower oil had higher polar forces compared to their dispersive components.

### 2.3. Interaction of Surfactants

Subsequent to the determination of single effects formed by each individual surfactant, synergistic properties of mixtures should also be evaluated. The interaction between the most effective surface agents are presented in Table 1 and Table 2. A 2k three-factorial design setup was applied, in which each factor took two levels (low and high). These levels were determined according to the results presented in Figure 3 and Figure 4. The lowest value was the lowest concentration of the component which had statistical decrease in surface tension values. Similarly, the highest value was taken as the concentration where the surfactant saturation was reached; in other words, surface tension remained constant with increasing surfactant concentration.

As presented previously in Figure 4, the surface tension reduction effect of tween 40 and tween 80 were very similar. For this reason, the interaction of span 80 with each tween compound was analyzed separately in a small trial shown in Table 1. For statistical evaluations, the population distributions of low and high concentrations of tweens were compared among themselves. Results showed that there was not any significant difference between tween-40- and tween-80-added solutions (Mann–Whitney–Wilcoxon test, *p* > 0.05).

Since no significant difference between tween 40 and 80 was found, the interaction effects between oil, span 80, and tween 40 were examined more detailed in Table 2. Data transformation was applied because the data was positively skewed (right skewed distribution) to get normal distribution. Three-way ANOVA was run to examine the interaction effect between oil, tween 40, and span 80 concentrations on surface tension values. Results showed that all two-way as well as three-way interactions have significant effect (*p* < 0.05).

### 2.4. Emulsion Stability Measurements

#### 2.4.1. Emulsion Droplet Size Determination and Optical Evaluations

The effect of presence and concentrations of surfactants and sunflower oil on emulsion size distributions were investigated. The droplet sizes of sunflower oil and lecithin solutions are given as surface area mean diameter (Sauter mean) in Table 3. The effects of any noise, bubbles, or agglomerations at the higher end of the data range were removed with modification of the results. During the analysis in Mastersizer, the liquid sample was placed in a stirred sample cell, which was filled with demineralized water. The device took the sample automatically from the stirred cell and sent it to the analyzer beam. Hence, the measurement of surfactants such as tween 40, tween 80, and low concentrations of lecithin was impossible due to their dilution in the sample cell. Additionally, particle size distributions of span 60 and span 80 did not overlap during the measurements of the parallels. Therefore, the results were not shown in the table. Interestingly, almost all concentrations of span components formed two peaks with similar frequencies. Span solutions have larger particles (>10 µm), which indicated that they could not be successfully integrated into the emulsion alone, and the process should be improved.

Droplet sizes of lecithin solutions decreased significantly with increasing concentration (Kruskal–Wallis test, *p* < 0.05). However, the reduction was insignificant for droplet sizes of sunflower oil emulsions (Kruskal–Wallis test, *p* > 0.05).

**Figure 6:**
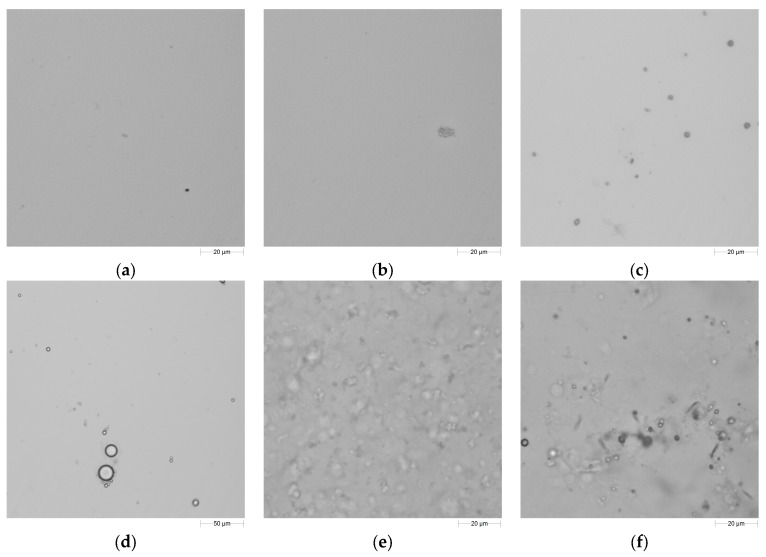
Microscope images of surfactant added coatings: (**a**) 0.25% tween 40; (**b**) 1% tween 40; (**c**) 0.25% span 80; (**d**) 1% span 80; (**e**) 0.25% lecithin; (**f**) 0.25% span 60. Surfactants listed in letters, incorporated into 1.25% sodium alginate + 2% glycerol solutions. Solutions were not diluted.

Solutions of 1.25% sodium alginate + 2% glycerol + surfactant (0.25–3.5%, 5 levels) were optically examined to determine the agglomeration, micelle formation, as well as the homogeneity of the coatings (Figure 6). Due to the water solubility of tweens, components were dissolved in the coating solution (Figure 6a). However, during optical evaluation of tween-added solutions (both tween 40 and tween 80), 10-µm-long gel-like particles were observed in the 0.5% and higher concentrations (Figure 6b). These structures were not detected in any 0.25% tween-incorporated samples. Sorbitan esters (spans) were not soluble in water, which could possibly cause the formation of particles with different sizes as seen in Figure 6c. In the images of higher span 80 concentrations (>1%), a translucent ring formation which surrounded the droplets could be observed (Figure 6d). Lecithin and span 60 produced carpet-like continuous structures which had an increasing intensity with increasing concentration (Figure 6e,f).

So far, the experiments were conducted to determine the individual effects of the components. According to the results obtained, three formulations were designed. All three formulations contained sunflower oil and span 80. Additionally, tween 40 and tween 80 were incorporated into second and third formulations, respectively.

**Figure 7:**
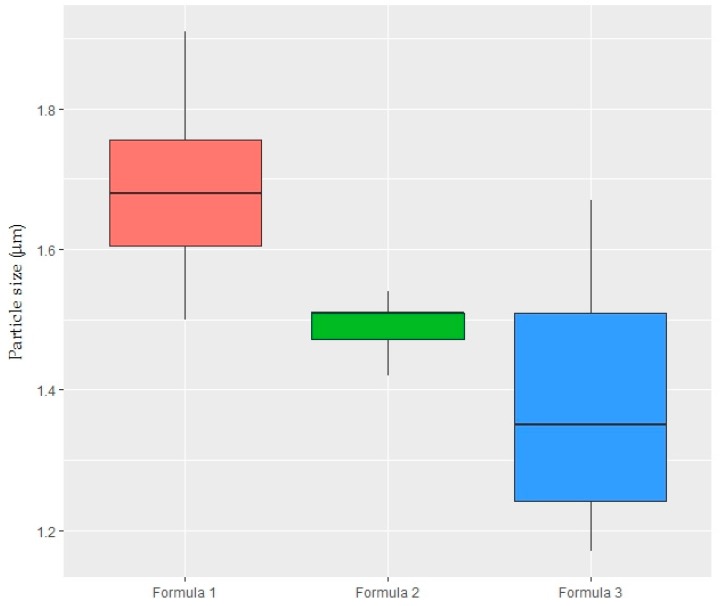
Box plot shows the emulsion droplet size (µm) as surface mean area diameter of the formulations. Formula 1: 1.25% sodium alginate + 2% glycerol + 0.2% sunflower oil + 1% span 80; Formula 2: 1.25% sodium alginate + 2% glycerol + 0.2% sunflower oil + 1% span 80 + 0.2% tween 40; Formula 3: 1.25% sodium alginate + 2% glycerol + 0.2% sunflower oil + 1% span 80 + 0.2% tween 80 (*n* = 6). Medians are shown with lines across the boxes. Minimum and maximum values (excluding outliers) are represented by whiskers (*n* = 3 × 2).

Droplet sizes of the created formulations are presented in Figure 7. Formulation 1 (oil + span 80) and Formulation 3 (oil + span 80 + tween 80) were found significantly different (one-way ANOVA, TukeyHSD test, *p* < 0.05). Addition of sunflower oil into the span 80 solution increased its reproducibility.

**Figure 8:**
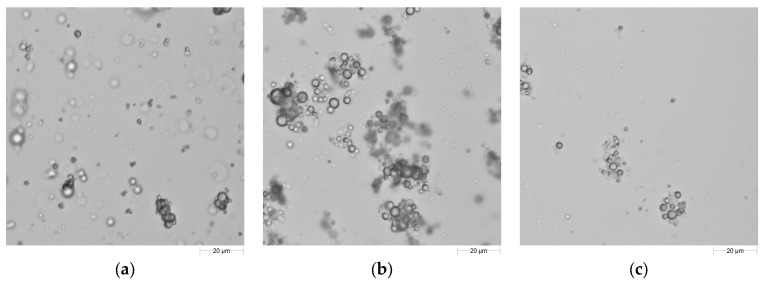
Digitized microscope images obtained from different formulations: (**a**) Formula 1: 1.25% sodium alginate + 2% glycerol + 0.2% sunflower oil + 1% span 80; (**b**) Formula 2: 1.25% sodium alginate + 2% glycerol + 0.2% sunflower oil + 1% span 80 + 0.2% tween 40; (**c**) Formula 3: 1.25% sodium alginate + 2% glycerol + 0.2% sunflower oil + 1% span 80 + 0.2% tween 80. Solutions were not diluted.

Formulations, which were designed based on surface tension and droplet size results, were also examined under microscope (Figure 8). All solutions had agglomerations, and agglomerations in tween 40-added solutions were greater in size and amount compared to the others.

#### 2.4.2. Creaming Index

Stability of the formulated emulsions was monitored for 26 h (*n* = 3). Phase separation or creaming was not observed in any test group.

### 2.5. Wettability

The wettability parameter should also be taken into account during optimization and comparison of the coating solutions. Superficial characteristics were measured on a low-energy strawberry surface. Table 4 summarizes surface tension, contact angle, adhesion coefficient (work of adhesion per unit area), cohesion coefficient (work of cohesion per unit area), and the wettability (spreading coefficient) data for the designed coating formulations determined above.

The third formulation (with tween 80) had the highest amount of work of adhesion, which caused the spreading of the coating on the surface (Welch test, Games–Howell post hoc test, *p* < 0.05). Despite that, work of cohesion, which induced the contraction of the coating, was lowest in first formulation (only with span 80). The highest wettability was achieved with first formulation (Welch test, Games–Howell post hoc test, *p* < 0.05). Nevertheless, wettability values were very close to each other.

## 3. Discussion

Surface tension is one of the principal representatives to characterize a surfactant and has been used to measure adsorption phenomenon of the coating solution [19]. Since we formulate the edible coating for hydrophobic surfaces such as surfaces of fruits and vegetables with natural protection layer, reduction of the surface tension of the coating solution is crucial for our study.

In the present work, the effects of the components on surface tension characteristics were assessed one after another, starting with the base material (sodium alginate). Therefore, the formulation has been gradually developed. By this means, their standalone effect as well as their interaction with the other components were observed.

The 3.5% sodium alginate was determined as the highest alginate concentration due to the rapid increase in the viscosity. Alginate did not change the surface tension values significantly, which indicated that the component did not have any adsorption activity in the liquid–vapor interface. Since the coating thickness of the deposited liquid on the food product increased proportionally with increasing viscosity [38,39,42], 1.25% alginate solution with 105.67 ± 1.63 mPa∙s viscosity was selected as a gelling structure of the solution.

Glycerol and sorbitol did not modify the surface tension of the liquids. Additionally, the results showed that there was no significant difference between the glycerol and sorbitol results. Only 20% glycerol concentration caused statistically different results in surface tension measurements. Rodríguez, et al. [43] also observed that starch and glycerol did not affect the surface tension of the solutions.

Addition of an oil source to alginate-based coating has many advantages: reducing moisture content, water transmission, and permeability of the coating [44], and being a solvent for oil-soluble surfactants. The results showed that sunflower, olive, and rapeseed oils also decreased the surface tension even in low concentrations such as 0.2%. Higher concentrations did not cause any further reduction of surface tension. Bearing in mind that coating formulation would be designed for fresh-cut fruits and vegetables, the addition of higher oil concentrations would also be disadvantageous in terms of consumer acceptance due to increasing calorie and energy uptake. Furthermore, the size of the bubbles increased with increasing oil concentrations, which would cause a creaming effect and phase separation. Designing a transparent, clear coating is beneficial in terms of higher acceptance and usability in a larger range in the food industry.

Curve fitting and their mathematical functions were constructed to have better data visualizations. In the present study, the smooth functions, which fit the data quite well (R^2^ ≥ 0.87), were defined to be used for interpolation for the data points to infer values where no data are available.

The highest surfactant concentration used in the study is 5% due to observing no decrease in surface tension with increased concentration. Also, Rodríguez, Osés, Ziani and Maté [43] emphasized that surfactants (for soy lecithin, tween 20, and span 80) concentrations above 5% do not allow to produce a uniform starch-based edible film. Porter [19] additionally explained that viscosity of the coating solution increased drastically at higher surfactant concentrations after reaching saturation, which led to the production of a gel-like structure due to the formation of lamellar or cylindrical micelles.

Surfactants formed similar decreasing curves with different slopes [y = (a + cx)/(1 + bx)]. Differences in the slope arose from the different sizes and shapes of hydrophobic and hydrophilic groups of the surfactants [19]. Without exception, surface tension values decreased rapidly as the concentrations of the surfactant increased until certain points (~1%, *w*/*w*). Decrease of surface tension slowed down at higher concentrations. This phenomenon was explained with the adsorption characteristics of surfactants by Porter [19]. When low concentration of surfactant was added to a solution, the majority of the surfactant molecules were adsorbed on the air–liquid interface, and by increasing the surfactant concentration, they continued to be adsorbed at the surface. This situation would continue until the saturation point, and when the saturation was reached, the surface tension became almost constant. After this point, increasing surfactant concentration would not decrease the surface tension, while the surfactant molecules would remain in the bulk of the solution. The collected information with optical evaluations was consistent with this theory. The differences in the surface tension curves (Figure 4) of surfactants could be elucidated with optical evaluations (Figure 6). When the surfactant solutions, which had statistically lower surface tension results, were examined under microscope, very few particles could be detected in the bulk solution. On the contrary, numerous particles with bigger sizes could be detected in surfactant solutions with higher surface tension results. This difference was especially noticeable in span 80 and span 60.

The results of tween 40 and tween 80 correlate favorably with Wan and Lee [45], who studied the effect of various polysorbates (tweens) on the surface tension, despite the fact that researchers found a slightly higher reduction in tween 40-added samples compared to tween 80. However, Ribeiro, Vicente, Teixeira and Miranda [27] found that the saturation point of tween 80 was 0.02% (*w*/*v*) in carrageenan solution, which was very low compared to our study. Rodríguez, Osés, Ziani and Maté [43] showed that span 80 was more effective than lecithin or tween 20 to reduce surface tension of starch-based edible films.

During solution preparation, span components caused foam formation on the top of the solution. Foam formation was more intense in span 60 compared to span 80. Thick foam formation on the surface of the coatings was explained in the literature in that these surfactants were strongly adsorbed at the air–liquid interface [19]. Figure 6c,d confirms with this theory. Even at the high concentration (≥1%), span 80 could not be optically detected in the solution. However, the same theory could not be verified for span 60 (Figure 6f). As observed in the photographs taken from different concentrations, span 60 particles were embedded in a translucent, gel-like structure, which did not accumulate on the liquid–air interface, but was found in high amounts in the bulk solution. The melting of span 60 during coating preparation process could be a reason for the formation of these structures.

Span 60 and span 80 can be distinguished from each other by the length and structure of their hydrocarbon chains; span 80 has double bonding in its acyl chain, while span 60 has a longer chain without any double bonding [46]. The surface tension difference of span 60 and span 80 could be caused by the double bond in the molecular structure of span 80, which decreases the hydrophobic nature of the surfactant [47]. In addition to that, the arrangement ability of the longer hydrocarbon chain in span 60 causes smaller surface area per molecule compared to span 80, which has larger molecular areas [47].

Determination of the polar and dispersive parts of liquid is not a straightforward process. Subsequent to the measurement of surface tension values, the dispersion force component of the liquid can be calculated with the help of a solid that has a completely nonpolar surface, such as PTFE film [33]. Dipole-dipole and hydrogen bonding interactions are polar interactions; Van der Waals type of interactions are dispersive interactions [48]. On the condition that only dispersion forces operate, the liquid or solid is nonpolar [30]. In the present study, coating was intended to design for fruits and vegetables, both fresh-cut and intact products with hydrophobic (in other words, nonpolar) surfaces. Therefore, relatively nonpolar liquids with higher dispersive and lower polar components would serve better as coating material. Tween 40, tween 80, and span 80 suited well to these circumstances (Figure 5) due to having relatively higher dispersive and lower polar forces.

The effects of tween 40 and tween 80 in mixtures of sodium alginate, glycerol, oil, and span 80 were compared in Table 1. It was apparent from the table that there was no significant difference between the same amount of tween 40- and tween 80-incorporated samples. Hence, both can be used in the edible coating formulation.

The interaction effects between sunflower oil, span 80, and tween 40 were identified in more detail in Table 2. The results correlated well with previous findings in Figure 3 and Figure 4. Concentration increase of all three components significantly reduced the surface tension. Span 80 had the highest reduction effect, followed by tween 40, and sunflower oil. The two-way and three-way interactions had significant effect on surface tension, which indicated that there were synergistic effects between surface active components.

Droplet size is an important agent of emulsion stability and, additionally, it affects many characteristics of solution such as viscosity, texture, and optical appearance [2,36]. In droplet size determination experiments, the focused concentration area was determined as 0–1% surfactant concentrations since the major surface tension decrease occurred within this concentration range (Figure 3 and Figure 4). In contradiction with earlier findings of Fernandez, André, Rieger and Kühnle [36], which stated that the type of droplet size distribution changed with concentration, in the present study, concentration increase did not cause a drastic change in droplet size distribution for the same type of surfactant. Distribution type changed only with surfactant type.

It has been suggested that droplet sizes between 0.01 and 10 µm were suitable emulsions. Droplet sizes smaller than 1 µm were referred to as molecular dispersions, while those larger than 1 µm were considered as coarse dispersions [49]. According to this definition, tweens and soy lecithin formed true solutions (molecular dispersions); on the other hand, spans and oils generated coarse dispersions. Translucent rings were formed around the span 80 droplets (Figure 6d). This ring could cause a scattering effect during the measurements in laser diffraction system, which could have affected the results of droplet size determination experiment.

It was very interesting that, oil and span mixtures had smaller particle sizes than oil and span formed alone, respectively, in alginate–glycerol solutions (Table 3 and Figure 7). Furthermore, particle sizes were significantly decreased with the addition of tween 80 to the system (Figure 7).

Aggregates of different sizes were embedded into the bulk solution of designed formulations (Figure 8a–c). Captured images showed that tween 80 could be incorporated successfully into the formulation and result in smaller aggregates with less intensity in the bulk solution.

Emulsions can have various instabilities, which cause creaming behavior. Considering the commercial importance of edible coating, visual creaming behavior was monitored as a function of storage hours. Since the designed solutions would be used for coating material, and would not be stored for long time, creaming was monitored only for a day, not longer. No phase separation was observed during this time interval.

Wettability is one of the important phenomena that have a strong impact on the effectiveness of formulated edible coating on food products [25,50]. The highest wettability result (−17.77 ± 2.29 mN/m) was achieved by Formulation 1 (1.25% sodium alginate + 2% glycerol + 0.2% sunflower oil + 1% span 80). However, considering the particle size, tween 80-incorporated Formulation 3 (1.25% sodium alginate + 2% glycerol + 0.2% sunflower oil + 1% span 80 + 0.2% tween 80) with −21.14 ± 4.64 mN/m wettability could also be successfully used. Ribeiro, Vicente, Teixeira and Miranda [27] found wettability on strawberry surfaces as −44.61 ± 3.05, −45.28 ± 0.88, and −38.89 ± 2.83 for starch-, carrageenan-, and chitosan-based formulated coatings, respectively.

## 4. Materials and Methods

### 4.1. Materials

Sodium alginate (Manugel GHB, FMC Biopolymer Co., Philadelphia, PA, USA), glycerol (Sigma-Aldrich Chemie GmbH, Steinheim, Germany), sorbitol (Acros Organics, Geel, Belgium), sunflower oil, olive oil, colza (rapeseed) oil (Rewe Bio, Rewe Markt Gmbh, Köln, Germany), tween 40 (polyoxyethylenesorbitan monopalmitate) (Sigma-Aldrich Chemie GmbH, Steinheim, Germany), tween 80 (polyoxyethylenesorbitan monooleate) (Sigma-Aldrich Chemie GmbH, Steinheim, Germany), span 80 (sorbitan monooleate) (Sigma-Aldrich Chemie GmbH, Steinheim, Germany), span 60 (sorbitan monostearate) (Merck KGaA, Darmstadt, Germany), and lecithin (made from GMO-free soybeans) (Carl Roth GmbH Co. KG, Karlsruhe, Germany), were used in coating formulations. Glycerol and sorbitol were employed as plasticizer. Tween 40 (hydrophilic–lipophilic balance, HLB = 15.6), tween 80 (HLB = 15.0), and span 80 (HLB = 4.3) were viscous liquid formed surfactants, while span 60 (HLB = 4.7) was in beige flakes and soy lecithin (HLB = 8.0) was in light brownish powder form.

Polytetrafluoroethylene (PTFE) (thickness: 500 µm) was purchased from SAHLBERG GmbH and Co. KG (Feldkirchen, Germany).

Fresh strawberries (*Fragaria ananassa*) were purchased from a local market (Freising, Germany). Samples were carefully checked to ensure uniform size and absence of any defects. Before measurements, samples were left at ambient temperature (~21 °C). Samples were cut into rectangular shapes (~3 cm × 2 cm) to reduce the slope of the surface.

### 4.2. Preparation of Coating Solutions

The amounts of components (i.e., gelling agent, plasticizers, oils, surfactants) were adjusted to achieve the specified amount in 100 g final solution.

Sodium alginate (0–3.5% (*w*/*w*), 9 levels) was dissolved in hot distilled water at 70 °C with continuous stirring (magnetic stirrer (500 rpm)) until complete dissolution was achieved and a clear solution was obtained. Plasticizers (glycerol and sorbitol (0–20% (*w*/*w*), 6 levels) were added to the solutions. Subsequently, surfactants (tween 40, tween 80, span 60, span 80, lecithin (0–5% (*w*/*w*), 11 levels) and vegetable oils (sunflower oil, olive oil, rapeseed oil (0–5% (*w*/*w*), 10 levels) were incorporated into the formulations. Hydrophobic surfactant (Span 60) was prepared according to the previous study of Villalobos et al. [51]. Span 60 was melted at 60 °C in distilled water with continuous stirring and added into the solution. The final weight of the emulsion increased to 100 g upon adding distilled water, and the mixture was continuously stirred with a magnetic stirrer to achieve dissolution of the surfactants. Afterwards, the mixtures were homogenized and emulsified using an ultra-turrax homogenizer (Miccra D-8, ART modern Labortechnik GmbH, Müllheim, Germany) at 10,500 min^−1^ for 5 min. The solutions were put in ultrasonic bath (Transsonic 460/H, Carl Roth GmbH Co. KG, Karlsruhe, Germany) at a frequency of 35 kHz for 5 min.

### 4.3. Surface Tension Measurements

Surface tension (γ_L_) of coating solutions was measured at room temperature (~21 °C), using the pendant drop method and Laplace–Young equation [28,29,52] with a drop shape analyzer (DSA1 v1.90, Kruss GmBH, Hamburg, Germany). Characteristic “pear shape” droplets were formed with a 500 µL syringe (Hamilton, Switzerland) and 1.991 mm needle (Kruss GmbH, Hamburg, Germany). Three replications were prepared for each solution and 10 measurements were taken on each replication.

Surface free energy (γ_S_) of PTFE film was determined by the sessile drop technique by a drop shape analyzer. For this purpose, water for chromatography (Merck KGaA, Darmstadt, Germany), diiodomethane (Sigma-Aldrich Chemie GmbH, Steinheim, Germany), and ethylene glycol (Sigma-Aldrich Chemie GmbH, Steinheim, Germany) were used as test liquids by placing 15 droplets of each test liquid on the PTFE film. Photographs were taken no longer than 5 s after surface–liquid contact. Surface tension values of the test liquids was given in the previous study of Senturk-Parreidt, Schmid, and Hauser [53]. The OWRK method was used for the calculation of γ_S_ of the PTFE film.

Determination of the polar and dispersive components of surface tension of liquid was carried out on PTFE film by using sessile drop arrangement with tangent method. Three replications were prepared for each solution and 10 measurements were taken on each replication.

### 4.4. Wettability Measurements

Wettability measurements were conducted according to the set up developed by Senturk-Parreidt, Schmid, and Hauser [53]. Droplets of 3 µL of the coating solutions were manually placed on strawberry epicarp, using a micropipette, which were kept perpendicular to the surface. Photographs were taken no longer than 5 s after surface–liquid contact. Twenty droplets of each coating solution were dispersed on the surface of the strawberry. Contact angle values of the droplets were measured with using ImageJ software [54] with the DropSnake plugin [55]. Adhesion coefficient (W_a_), cohesion coefficient (W_c_), and spreading coefficient (W_s_) were calculated as stated in the previous studies of Ribeiro, Vicente, Teixeira and Miranda [27] and Casariego, Souza, Vicente, Teixeira, Cruz and Díaz [50]. The derived equations can be summarized as:

W_a_ = γ_SV_ + γ_LV_ − γ_SL_ = γ_LV_ × (1 + cosθ) (1)

W_c_ = 2 × γ_LV_ (2)

W_s_ = W_a_ − W_c_ (3)

### 4.5. Viscosity Measurements

Physica MCR 301 rheometer (Anton Paar GmbH, Graz, Austria) using a measuring system (CC27) with measuring cup (diameter 28.913 mm) and measuring bob (diameter 26.656 mm and length 40.025 mm) was used to measure the dynamic viscosity of the approximately 15 mL aliquots of coating solution at room temperature (~21 °C). Three measurements were performed for each solution with constant shear rate.

### 4.6. Emulsion Stability Measurements

#### 4.6.1. Droplet Size Measurement with Laser Diffraction System

Three replications of 0.25–3.5% (5 levels) surfactants/oil-added 1.25% alginate + 2% glycerol samples were prepared. The air, which was present in the solutions, was removed with vacuum application. For this purpose, solutions were transferred into 20 mL glass vials and placed in an airtight vacuum chamber (designed by Fraunhofer Institute for Process Engineering and Packaging IVV, Freising, Germany) connected to a vacuum pump (N740, 40 L/min flow rate and 10mbar absolute vacuum, KNF Neuberger GmbH, Freiburg, Germany) and digital vacuum/barometer (GDH 200-14, Greisinger Electronic GmbH, Regenstauf, Germany).

The droplet size measurement was carried out with a Malvern Mastersizer S long bench model MSS, Software version 2.19, with the small sample dispersion unit MS 1 (volume max. 150 mL) and the 300 mm RF lens (Malvern Instruments Ltd., Worcestershire, UK). For the calculation of the droplet size, a polydispersity distribution was chosen as analysis model, and Mie Theory with the optical density for the wet phase, 1.33, and for the disperse part, 1.46, were set (Software Model: 3NHD). To arrange the sample concentration, the obscuration, which denotes the amount of laser light that has been lost by passing through the sample, was adjusted between 10% and 30%. The measurement was started after 2–3 min of dispersion time. The mean values of the droplet sizes were calculated as mean of three samples which were measured twice.

#### 4.6.2. Optical Evaluation

Samples, which were prepared for droplet size measurements (0.25–3.5%, 5 levels) were used also in optical evaluations. A droplet was prepared between glass slides and examined in Morphologi G3 S microscope model 2410 (magnification: ×20 and ×50) with Software 8.11 (Malvern Instruments Ltd., Worcestershire, UK). Diascopic light was used with an intensity of 70–80%. The device can measure the particle size from 0.5 µm to 1000 µm. Optical microscope images with length scale were taken.

#### 4.6.3. Creaming Index

Immediately after preparation, 25 mL coating solutions were placed in transparent, graduated cylindrical plastic tubes (diameter: 25 mm, height: 110 mm) and sealed with their plastic lids. Three replications were prepared for each coating solution. After a gentle agitation, tubes were left for 26 h at room temperature (~21 °C) without moving. Photos were taken at 0, 6, 12, 20, and 26 h with Nikon D3300 digital camera (Sendai Nikon Co., Tokyo, Japan) and Tokina 100 mm F2.8 Macro lens (Kenko Tokina Co., Ltd., Tokyo, Japan). The distance between the camera and the tubes was fixed for all measurements at 18.5 cm. Creaming was characterized by calculating creaming index (CI):

(4) $$\mathrm{CI}\;(\%)\;=\;100\;\times \;\frac{H_{S}}{H_{E}}$$

where *H_S_* is the height of serum layer and *H_E_* is the total height of the emulsion [37].

### 4.7. Statistical Evaluations

Means and standard deviations were performed with Microsoft Excel 2010 (Microsoft Corp., Redmond, WA, USA). Graphics, statistical evaluations, etc. were performed with an open source program, R 3.3.2 for Windows. ggplot2 [56], grid [57], gridExtra [58], plyr [59], graphics [57] extrafont [60] packages for graphics; car [61], lsr [62], userfriendlyscience [63] packages for statistical analysis were used. One dimensional roots were found with rootSolve package during determination of dispersive components of liquid surface tensions [64,65]. The best fitting curves and their functions were determined with Table Curve 2D v5.01 (Systat Software Inc., San Jose, CA, USA).

## 5. Conclusions

In the present study, the effects of various coating components, as well as their concentrations on surface tension, and emulsion droplet size were investigated in order to design an effective edible coating with high wettability on hydrophobic nonpolar food surfaces. The results showed that addition of different sodium alginate and plasticizer (i.e., glycerol and sorbitol) concentrations did not alter the surface tension results. However, vegetable oils (i.e., sunflower, olive, rapeseed oils) and surfactants diminished the surface tension.

Surfactants are the most important factor affecting surface tension, and in this way the wettability of the coating solution on food products. The presence, type, and concentration of surfactants affected surface tension, size, as well as distribution of emulsion droplets differently. Span 80, tween 80, and tween 40 were found as the most effective surface active agents, respectively.

As a compromise between achieving maximum reduction of surface tension and using minimum amount of coating component, the use of 1.25% sodium alginate, 2% glycerol, 0.2% sunflower oil, and 1% span 80 in the formulation was recommended. The results previously presented have led us to conclude that the addition of tween 80 into the formulation decreased the droplet size and amount in the bulk solution.

This study provided an important methodology for the edible coating/film formulators. The findings might have many implications for edible coating/film research and industry applications. The constructed curves and functions enables coating formulators to conduct interpolation for the data points to infer values where no data are available. Further studies may concentrate on investigating the effects of suggested formulations on achieving uniform coating, coating thickness, and transport mechanisms.

## Figures and Tables

**Table 1:** Interaction effect of surfactants on surface tension (γ_L_) values of coating solutions. Each formulation (each row) was incorporated into 1.25% sodium alginate + 2% glycerol + 0.05% sunflower oil solutions (*n* = 3).

Span 80 (%)	Tween 40 (%)	Tween 80 (%)	γ_L_ (mN/m)
0.06	0.03	-	55.42 ± 0.66 ^A^
0.06	-	0.03	54.64 ± 0.14 ^A^
1	-	1	38.05 ± 0.30 ^B^
1	1	-	37.75 ± 0.27 ^B^

For each formulation, different superscripts in column are significantly different (*p* ≤ 0.05).

**Table 2:** Interaction effect of components on surface tension (γ_L_) values of coating solutions. Each formulation (each row) was incorporated into 1.25% sodium alginate + 2% glycerol solutions (*n* = 3).

Sunflower Oil	Span 80 (%)	Tween 40 (%)	γ_L_ (mN/m)
0.05	0.06	0.03	55.42 ± 0.66 ^A^
1	0.06	0.03	50.10 ± 0.25 ^B^
0.05	0.06	1	43.72 ± 0.42 ^C^
1	0.06	1	43.13 ± 0.05 ^C^
0.05	1	0.03	33.63 ± 0.60 ^D^
1	1	0.03	34.99 ± 0.28 ^E^
0.05	1	1	37.75 ± 0.27 ^F^
1	1	1	37.16 ± 0.12 ^F^

For each formulation, different superscripts in column are significantly different (*p* ≤ 0.05).

**Table 3:** Surface area mean diameter (Sauter mean) of droplets of components at specified concentrations. Each component was incorporated into 1.25% sodium alginate + 2% glycerol solutions (*n* = 3 × 2).

Component	0.25%	0.5%	0.75%	1%	3.5%
Sunflower oil	2.31 ± 0.09 _a_	2.08 ± 0.21 _a_	2.08 ± 0.30 _a_	2.06 ± 0.16 _a_	2.06 ± 0.43 _a_
Lecithin	- ^1^	- ^1^	0.53 ± 0.01 _b_	0.44 ± 0.01 _c_	0.41 ± 0.01 _d_

^1^ Measurements cannot be performed due to the dilution of the samples in sample cell of the Mastersizer. or each component, different subscripts in rows are significantly different (*p* ≤ 0.05).

**Table 4:** Surface tension (γ_L_), contact angle θ (°), adhesion coefficient (W_a_), cohesion coefficient (W_c_), and wettability (W_S_) data of formulations. Formula 1: 1.25% sodium alginate + 2% glycerol + 0.2% sunflower oil + 1% span 80; Formula 2: 1.25% sodium alginate + 2% glycerol + 0.2% sunflower oil + 1% span 80 + 0.2% tween 40; Formula 3: 1.25% sodium alginate + 2% glycerol + 0.2% sunflower oil + 1% span 80 + 0.2% tween 80 (*n* = 20).

Formulation	γ_L_ (mN/m)	θ (°)	W_a_ (mN/m)	W_c_ (mN/m)	W_S_ (mN/m)
Formula 1	31.80 ± 0.09 ^A^	66.39 ± 5.98 ^D^	45.83 ± 2.29 ^F^	63.60 ^H^	−17.77 ± 2.29 ^L^
Formula 2	36.26 ± 0.51 ^C^	72.62 ± 5.31 ^E^	47.05 ± 3.05 ^FG^	72.52 ^K^	−25.47 ± 3.18 ^N^
Formula 3	35.41 ± 0.27 ^B^	65.99 ± 8.28 ^D^	49.68 ± 4.64 ^G^	70.82 ^I^	−21.14 ± 4.64 ^M^

For each variable, different superscripts in columns are significantly different (*p* ≤ 0.05).
